# Outcomes of patients with de novo and secondary acute myeloid leukaemia treated with front‐line hypomethylating agents and venetoclax: A retrospective Italian study

**DOI:** 10.1111/bjh.70587

**Published:** 2026-05-28

**Authors:** Ilaria Tanasi, Edoardo Tamellini, Elena Marchetti, Eleonora De Bellis, Cristina Ancora, Francesco Angotzi, Davide Facchinelli, Francesca Capraro, Giulia Battaglia, Alessandra Sperotto, Michele Gottardi, Matteo Leoncin, Mauro Krampera, Mario Tiribelli, Carmela Gurrieri

**Affiliations:** ^1^ Hematology and Bone Marrow Transplant Unit, Section of Biomedicine of Innovation, Department of Engineering for Innovative Medicine University of Verona Verona Italy; ^2^ UCO Ematologia Azienda Sanitaria Universitaria Giuliano Isontina Trieste Italy; ^3^ Hematology Unit Azienda Ospedale‐Università and University of Padova Padua Italy; ^4^ Department of Hematology San Bortolo Hospital Vicenza Italy; ^5^ Hematology Unit Azienda ULSS3 Serenissima, Ospedale dell'Angelo Venice Italy; ^6^ Division of Hematology and Bone Marrow Transplantation, Department of Medical Area University of Udine Udine Italy; ^7^ Onco Hematology Unit, Department of Oncology Veneto Institute of Oncology (IOV)‐IRCCS Padua Italy

**Keywords:** leukaemia, secondary acute myeloid leukaemia, venetoclax

To the Editor,

Secondary acute myeloid leukaemia (sAML), defined by a history of antecedent myelodysplastic syndrome (MDS) or myeloproliferative neoplasm (MPN), or the presence of myelodysplasia‐related gene mutations or cytogenetic abnormalities, has a worse prognosis compared to de novo acute myeloid leukaemia (dnAML) in patients receiving intensive treatment.^1^, ^2^ Evidence on the outcomes of sAML patients treated with hypomethylating agents and venetoclax (HMA‐VEN) remains limited.^2^, ^3^, ^4^, ^5^

This retrospective, multicentre study aimed to compare outcomes, response rates and response duration of adult patients with dnAML and sAML treated with HMA‐VEN.

We included 184 newly diagnosed adult acute myeloid leukaemia (AML) patients treated upfront with at least one cycle of HMA‐VEN across seven Italian centres between 2019 and 2024. The study was performed in accordance with the Declaration of Helsinki and was approved by the Institutional Review Board (protocol no 24919 of 26 April 2023). The diagnosis of AML was established according to the 2022 International Consensus Classification.^6^ sAML encompasses cases with myelodysplasia‐related cytogenetic or molecular abnormalities or with a prior history of MDS or MPN, irrespective of genetic features. Response assessment followed local standards, and responses (complete remission [CR], CR with incomplete haematological recovery [Cri], composite CR [CR + Cri], morphological leukaemia‐free state [MLFS] and partial response [PR]) were defined according to European Leukemia Net (ELN) criteria.^7^

Categorical variables were compared using Fisher's exact test, and continuous variables were compared using the Mann–Whitney *U*‐test. Survival analyses were conducted using the Kaplan–Meier method. Multivariate analysis was performed using a Cox proportional hazards regression model. Overall survival (OS) was defined as the time from the start of therapy to death or last follow‐up. Relapse‐free survival (RFS) was defined as the time from achievement of CR/CRi to relapse or death, whichever occurred first. Both OS and RFS were censored at the time of allogeneic haematopoietic stem cell transplant.

Overall, 93 (51%) of 184 patients were classified as dnAML and 91 (49%) as sAML. Twenty‐five of 113 patients (22%) initially defined as dnAML were reclassified as sAML due to cytogenetic or molecular features.

Median age at diagnosis was 75 years for both dnAML (range, 26–87) and sAML (range, 42–85) (*p* = 0.354). In the dnAML group, 38% of patients were male (*n* = 35) compared with 62% in the sAML group (*n* = 58, *p* = 0.0004).

Forty‐five dnAML patients (48%) were classified as AML not otherwise specified (NOS), 38 (41%) had recurrent genetic abnormalities and 10 (11%) had *TP53*‐mutated AML.

Among sAML patients, 51 (56%) had a history of MDS, and 18 of them (38%) were previously treated with azacitidine. Nine sAML patients (10%) had a diagnosis of blast‐phase MPN, while 64 patients (70%) had MDS‐related cytogenetic alterations (*n* = 31) or gene mutations (*n* = 33). Eight patients (9%) had tumor protein p53 (*TP53*)‐mutated AML, nine (10%) AML NOS and one patient had an Nucleophosmin 1 (*NPM1*)‐mutated AML.

As expected, adverse cytogenetics were reported in 15% of dnAML (*n* = 11/72) and 54% of sAML patients (*n* = 47/87) with available data (*p* < 0.001). Patients' baseline characteristics are summarized in Table 1.

**TABLE 1:** Baseline characteristics of patients with de novo and secondary AML.

Patients' characteristics	De novo AML (*n* = 93)	Secondary AML (*n* = 91)	*p* value
Median age (range)	75 (26–87)	75 (42–85)	0.354
Male sex, no. (%)	35 (38)	58 (62)	0.004
ECOG <2, no. (%)	61 (66)	55 (60)	0.541
Diagnosis			
AML NOS, no. (%)	45 (48)	9 (10)	<0.001
AML with recurrent genetic abnormalities, no. (%)	38 (41)	1 (1)	
AML with myelodysplasia‐related gene mutations, no. (%)	0 (0)	33 (36)	
AML with myelodysplasia‐related cytogenetic abnormalities, no. (%)	0 (0)	31 (34)	
AML with mutated *TP53*	10 (11)	8 (9)	
Blast‐phase MPN	0 (0)	9 (10)	NA
Prior history of MDS	0 (0)	51 (56)	NA
Prior HMA treatment	0 (0)	18/51 (35)	NA
2022 ELN risk category, no. (%)			
Favourable	21/73 (29)	0/89 (0)	<0.001
Intermediate	38/73 (52)	15/89 (17)	
Adverse	14/73 (19)	74/89 (83)	
2024 ELN risk category, no. (%)			
Favourable	16/32 (50)	29/46 (63)	0.752
Intermediate	6/32 (19)	7/46 (15)	
Adverse	10/32 (31)	9/46 (19)	
Treatment			
HMA type (azacitidine/decitabine), no. (%)	78/15 (84/16)	73/18 (80/20)	0.561
No. of cycles (range)	5 (1–50)	4 (1–54)	0.176
Allo‐SCT	7 (7)	7 (8)	0.812

Abbreviations: AML, acute myeloid leukaemia; dnAML, de novo AML; ECOG, eastern cooperative oncology group; ELN, European Leukemia Net; HMA, hypomethylating agent; MDS, myelodysplastic syndrome; MPN, myeloproliferative neoplasm; NA, not applicable; NOS, not otherwise specified; sAML, secondary AML.

The median number of cycles received was 5 for dnAML (range 1–50) and 4 for sAML patients (range 1–54).

Among 172 evaluable patients, an overall response rate (ORR) (CR + CRi + PR + MLFS) was observed in 84.9% of patients in the entire cohort. A significantly greater proportion of sAML patients were refractory to treatment compared with dnAML (21.8% vs. 8.2%, respectively; *p* = 0.018).

Median time to best response was 54 days for both groups (95% confidence interval [CI] of median 42–65 and 35–61 for dnAML and sAML respectively; *p* = 0.51).

Composite CR did not significantly differ between dnAML and sAML (81.2% vs. 70.1%, respectively; *p* = 0.111). CR was achieved in 61 (71.8%) of dnAML and 52 (59.8%) sAML patients (*p* = 0.110). Besides, 1‐year cumulative incidence of relapse (CIR) was higher in sAML compared to dnAML (58.2% vs. 39.9%, *p* = 0.07). Consequently, responses were more durable in dnAML, with a median RFS of 14 months (95% CI: 10–25 months) versus 9 months in sAML (95% CI: 5–14 months, *p* = 0.029; Figure 1). With a median follow‐up of 25 months (range 1–60 months), median OS was significantly lower for sAML than for dnAML (11 months, 95% CI: 9–14 months vs. 19 months, 95% CI: 13–25 months; *p* = 0.024; Figure 1).

**FIGURE 1:**
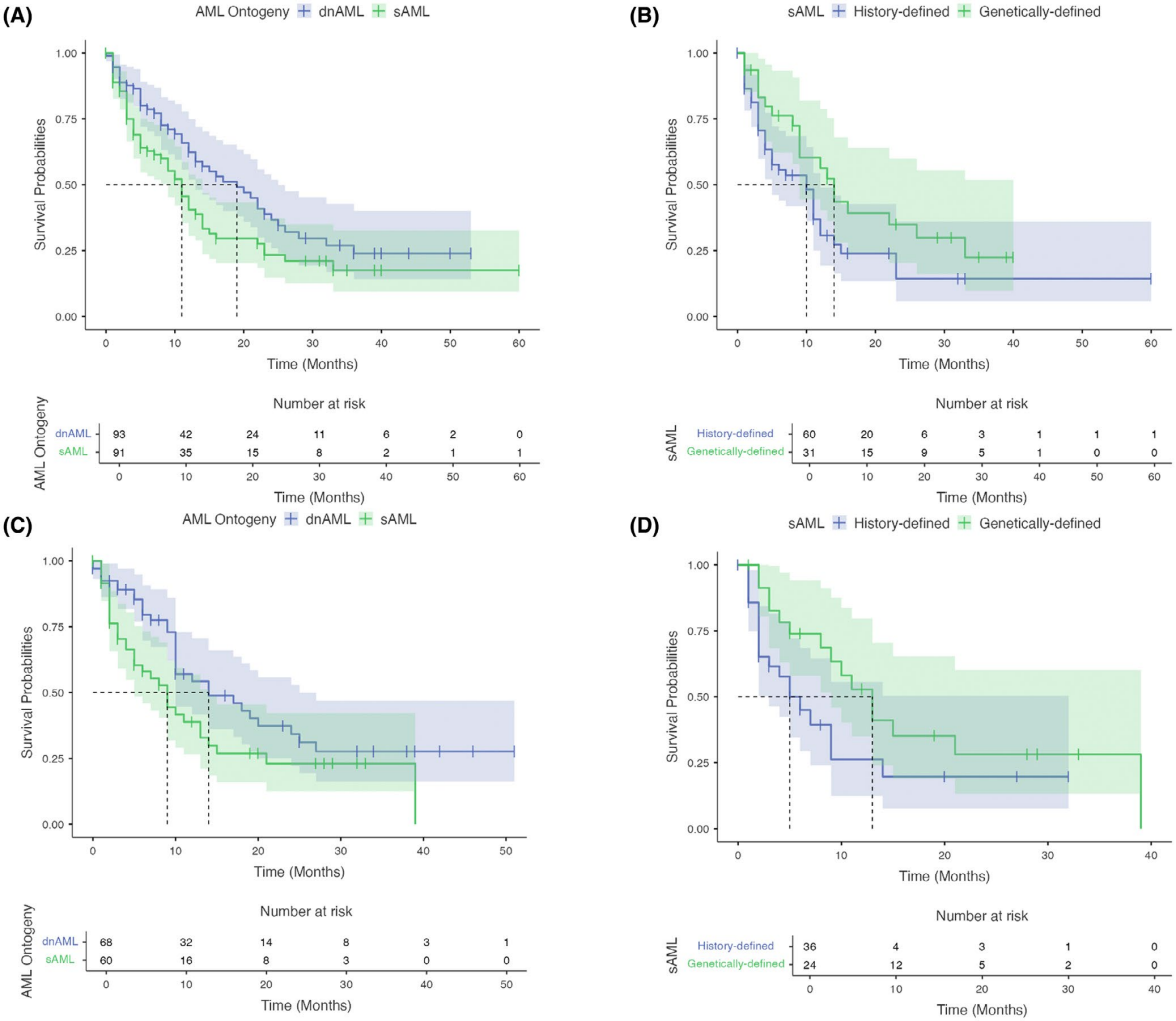
(A) Overall survival of patients with de novo (dnAML) and secondary AML (sAML); (B) overall survival of patients with sAML history‐defined and genetically defined; (C) relapse‐free survival of patients with dnAML and sAML patients; (D) relapse free survival of patients with sAML history‐defined and genetically defined. AML, acute myeloid leukaemia; SCT, stem cell transplantation.

When comparing outcomes based on ELN 2022 cytogenetic risk classification, non‐adverse cytogenetics were associated with a survival advantage only in dnAML (median OS: 25 vs. 10 months in sAML with non‐adverse cytogenetics, *p* = 0.002). Median OS was not significantly different between dnAML and sAML patients with adverse cytogenetics (6 vs. 12 months, respectively; *p* = 0.07).

Seventy‐seven patients with available data were stratified according to ELN 2024 risk classification. Median OS was not reached (NR) versus 14 months in the favourable risk group (*p* = 0.43), 10 vs. 11 months in the intermediate‐risk group (*p* = 0.9) and 6 vs. 4 months in the adverse risk group (*p* = 0.32) for dnAML and sAML respectively.

On multivariate analysis, male sex (HR 1.6, 95% CI: 1.1–2.5; *p* = 0.008), prior history of myeloid disorder (hazard ratio [HR] 1.9, 95% CI: 1.0–3.9; *p* = 0.031) and *TP53* mutations (HR 3.6, 95% CI: 1.6–7.8; *p* = 0.002) were the only variables independently associated with survival.

We also aimed to assess whether, within the sAML group, a prior history of MDS or MPN or the cytogenetic and molecular features had a greater prognostic impact. Therefore, we separately analysed the outcomes of patients with sAML and a prior history of MDS or MPN (history‐defined, *n* = 60) and those with AML‐related cytogenetic or molecular features (genetically defined, *n* = 31).

ORR and composite Complete Remission (cCR) rate were lower for history‐defined than genetically defined sAML patients (72% vs. 90% and 64.9% vs. 80%; *p* = 0.061 and 0.144 respectively).

The 1‐year CIR was higher in the history‐defined than in the genetically defined group (72% vs. 43.9%, *p* = 0.071), with a median RFS of 5 and 13 months respectively (*p* = 0.061; Figure 1). Besides, patients in the genetically defined sAML group had superior median OS than history‐defined sAML (14 months, 95% CI: 9 months–not reached vs. 10 months, 95% CI: 5–12 months, *p* = 0.081; Figure 1).

No significant difference in response rate and OS was observed between history‐defined sAML patients previously treated with azacitidine for MDS versus HMA‐naïve patients (cCR rate 44.4% vs. 69.2% and median OS 7 vs. 6 months; *p* = 0.127 and 0.721 respectively).

Furthermore, history‐defined sAML patients had poor outcomes regardless of cytogenetic risk (median OS 9 months with adverse cytogenetics vs. 10 months without; *p* = 0.63).

We also assessed the impact of *TP53* mutations on response rate and outcomes in both dnAML and sAML. Of 81 patients with known *TP53* status, 63 (78%) were wild type and 18 (22%) were mutated. Among 18 *TP53*‐mutated cases, 10 (56%) had dnAML and 8 (44%) had sAML.

Despite the small sample size, sAML patients with *TP53* mutations had a lower cCR rate (37%) than *TP53*‐mutated dnAML (60%) and *TP53* wild‐type sAML (79%; *p* = 0.637 and *p* = 0.039, respectively).

Median OS was similarly poor in *TP53*‐mutated patients with dnAML and sAML (7 vs. 4 months, respectively; *p* = 0.121). Nevertheless, sAML patients with *TP53* wild type had a significantly longer OS compared to *TP53*‐mutated sAML (*p* = 0.002), and a comparable outcome to dnAML (median OS 18 vs. 21 months, respectively; *p* = 0.679). We did not find any significant difference in OS and response rates among sAML patients carrying spliceosome mutations (*n* = 26) and those without (*n* = 18; *p* = 0.351 and 0.526 respectively).

Data on outcomes of sAML patients treated front‐line with HMA‐VEN remain scarce. In the registrational VIALE‐A trial, only a minority of patients had sAML (25%), and those previously exposed to HMA were not eligible.^5^

We therefore examined the efficacy of HMA‐VEN in routine practice, comparing outcomes between dnAML and sAML.

Although the ORR in the whole cohort reached 84.9%, disease ontogeny influenced prognosis. Responses in sAML were also frequent but less durable, and both RFS and OS were significantly shorter than in dnAML (9 and 11 vs. 14 and 19 months respectively). These findings are aligned with a recent prospective study, in which more than half of the cohort had sAML, showing that ontogeny was independently associated with OS.^4^

A distinctive aspect of our analysis is the way sAML was defined. Most published studies have not separated patients according to an antecedent documented myeloid neoplasm, independently of cytogenetic/molecular criteria. When we applied this distinction, patients with a ‘history‐defined’ sAML (i.e. prior MDS or MPN) fared substantially worse than those with a ‘genetically defined’ sAML (MDS‐related genetics without a known antecedent phase). In the former, we observed lower response rates (ORR 72%), a higher 1‐year relapse rate (72%) and a median OS of 10 months, while genetically defined sAML had responses and outcomes closer to dnAML.

This observation is consistent with recent molecular data suggesting that myelodysplasia‐related spliceosome mutations, historically viewed as strongly adverse in chemotherapy‐treated AML, may not exert the same negative impact in patients treated with HMA‐VEN.^8^, ^9^, ^10^

Overall, our findings suggest that, in the venetoclax era, sAML is not a biologically uniform entity and that patients with a documented antecedent myeloid disorder may derive less benefit from this treatment.

## AUTHOR CONTRIBUTIONS

**Ilaria Tanasi:** Conceptualization; methodology; data curation; investigation; formal analysis; supervision; writing – original draft. **Edoardo Tamellini:** Conceptualization; methodology; data curation; investigation; formal analysis; writing – review and editing. **Elena Marchetti:** Conceptualization; data curation; investigation; formal analysis; writing – review and editing. **Eleonora De Bellis:** Investigation; writing – review and editing. **Cristina Ancora:** Investigation; writing – review and editing. **Francesco Angotzi:** Investigation; writing – review and editing. **Davide Facchinelli:** Investigation; writing – review and editing. **Francesca Capraro:** Investigation; writing – review and editing. **Giulia Battaglia:** Investigation; writing – review and editing. **Alessandra Sperotto:** Investigation; writing – review and editing. **Michele Gottardi:** Investigation; writing – review and editing. **Matteo Leoncin:** Investigation; writing – review and editing. **Mauro Krampera:** Investigation; writing – review and editing. **Mario Tiribelli:** Methodology; investigation; writing – review and editing. **Carmela Gurrieri:** Methodology; supervision; investigation; writing – review and editing.

## FUNDING INFORMATION

The authors received no funding for this work.

## CONFLICT OF INTEREST STATEMENT

The authors have no conflicts of interest to disclose.

## Data Availability

The data that support the findings of this study are available from the corresponding author upon reasonable request.
